# Hæmorrhagia, or Hæmorrhage

**Published:** 1839-01-05

**Authors:** N. Chapman

**Affiliations:** Professor of the Theory and Practice of Physic, in the University of Pennsylvania


MEDICAL EXAMINER.

DEVOTED TO MEDICINE, SURGERY, AND THE COLLATERAL SCIENCES.

No. 1.] PHILADELPHIA, SATURDAY, JANUARY 5, 1839. [Vol. II.

### HÆMORRHAGIA, OR HÆMORRHAGE.

*By* N. Chapman, M. D., *Professor of the Theory and Practice of Physic, in the University of Pennsylvania.*

[Reported for this Journal.]

Conformably to its etymology, the term hæmorrhage embraces every effusion of blood, however induced. Five modes have been assigned in which it may take place, by rhexis or rupture of a vessel, by diaresis or division of it, by diabrosis or erosion of its coats, by diapedesis or transudation through them, and by anastomosis, or dilatation of the mouths of the exhalents, so as to allow the blood to escape.

Founded in part, on the supposed modes of its production, an arrangement was early made of hæmorrhage into traumatic, as occasioned by wounded vessels, and into spontaneous, when occurring without such vascular injuries. But more accurately expressive of the two distinctions, are the terms physical and vital, lately introduced, these, indeed, denoting with sufficient precision, the nature of the lesions on which the sanguineous emissions depend.

Custom having hitherto resigned the physical hæmorrhages to the province of surgery, I shall, in obedience to it, do the same,—limiting the ensuing inquiry, therefore, to the other description of the affection. Corresponding more exactly, in my opinion, with a just pathology of vital hæmorrhages than any previous classification of them, it is my intention to distribute the subject under the titles of *active*, less *active*, and *passive*, thereby indicating the several gradations of the hæmorrhagic states. The symptoms, of course, vary as connected with one or the other of these conditions. In the first, it happens that the effusion takes place, with little or no premonition, though, perhaps, as frequently, it is preceded by a full, and somewhat struggling pulse, heat and redness of surface, with a sense of congestion in the part whence the blood is to be eliminated. On other occasions, it presents more of the intermittent character, being ushered in by a chill, followed by fever, ceasing with the subsidence of this excitement, and reverting on a recurrence of it. The law of periodicity may be very strictly observed, in regard to the order of the paroxysms. Cases are recorded of hæmorrhage in all its locations, returning daily, every other day, and at more distant intervals. Generally, however, the febrile condition is steady, or, at least, there are continued manifestations of an increased force of the circulation, and undue local determinations.

The effects of hæmorrhage depend mainly, on the quantity of the effusion, the rapidity with which it takes place, the part whence it proceeds, and the capacity to bear the debilitating process. No one doubts the influence of the two first causes,—and, I think, that of the others is scarcely less apparent. Thus, a few ounces of blood from the nostrils, or the rectum, will sometimes induce syncope, or a disposition to it, and by even moderate repetitions of such bleedings in either case, for any length of time, depravations inevitably follow. Coming, on the contrary, from the mucous tissue of the lungs or the uterus, though a much larger amount be lost, there are no such results immediate, or remote, and certainly, not to the same extent. Equally is it true, that while some persons will sustain an immense loss without any ostensible injury, others instantly sink under very slight effusions, or have entailed a lengthened suffering,—and this shall happen where no material difference between them is discernible in vigour or integrity of constitution. The same disparity, in this respect, may be observed in relation to the effect of venesection, or topical bleeding, ascribable altogether, as in the former instance, to some idiosyncracy not always intelligible. Many instances to this purport, have I seen.

In active hæmorrhage, however, where the flow is moderate, and escapes through the natural passages, great relief is afforded to the existing symptoms, and a renewal of the hæmorrhage usually prevented. But when the loss of blood is disproportioned to the exigency, and, of course, still more, when exceedingly profuse, or rapidly emitted, the action of the heart and vessels is so reduced as scarcely to be recognised,—the surface becomes cold, collapsed, and pallid, the respiration quick and embarrassed, chills or rigours take place, followed by distraction of the senses, dimness of vision, ringing in the ears, with nervous tremors, some cerebral confusion, and, ultimately, syncope, or, perhaps, fatal convulsions. Yet after a while, reaction commonly ensues, attended by a quick, hard, irritated pulse, alternate chills and flushes, or a more permanent and unequal distribution of temperature, and, very often, by repetitions of the hæmorrhage to a greater or less extent. Extreme perturbation, however, of body and mind, is sometimes now betrayed,—the countenance becomes florid or pale in rapid succession, the tongue furred, the skin hot and dry, the whole circulatory machinery acts anormally,—and the pulse simulates by its impetus, its fulness, and even its tenseness, the real synochal state.

Enduring in this way for any length of time, the consequences of the hæmorrhage are exceedingly serious, amounting nearly to an universal disorder of the functions of the animal economy. The aspect is that of confirmed and inveterate dyscracy, pale, sallow, or livid in patches—sometimes bloated, particularly the face—the circulation being accelerated and feeble, though apt still to be tumultuous on any mental or corporeal agitation. Cerebral excitement, indicated by intolerance of light or sounds, or by slight delirium, is indeed very common.

Not, arrested, the case rapidly advances with an aggravation of the foregoing symptoms. To increased exhaustion, there are added greater embarrassments of breathing, sluggishness of body, obtuseness of mind, drowsiness, more violent throbbings of the arteries, and palpitations of the heart on any exertion or emotion, with considerable irritation in the mucous lining of the alimentary and pulmonary passages, deficient urination; and, where death does not promptly take place, dropsy of the cellular tissue, or of the cavities, or of both, results, which more slowly leads to the same catastrophe.

Much of this account is applicable to the *less active* hæmorrhages. They differ, however, materially in their early symptoms. Connected from the beginning with a reduced, attenuated, and frequently depraved state of the system, and rarely preceded or accompanied by any of the signs of the *motimen hæmorrhagicum*, the train of secondary phenomena I have noticed are almost constantly evinced by any undue loss of blood, and hurry on with more rapid speed to the final termination which has been described.

Nothing scarcely need now be said of *passive* hæmorrhages, since the circumstances in which they occur, and the mode of production to be immediately detailed, are calculated to shed sufficient light on them at present, without any formal recital of their history.

As might be conjectured, they are mostly the effect of the lowest state of vitality, whether induced by acute or chronic distemperatures,—and occurring in such general pravities of system, are usually very pervading, the effusions coming from several parts, or indeed, as I have seen, from nearly the whole body, simultaneously. Lucan says of them:

> "Sanguis erant lacrymæ: quacunque foramina novit

> Humor, ab his largus manat cruor: ora redundat,

> Et patulæ nares: sudor rubet: omnia plenis

> Membra fluunt venis: totum est pro vulnere corpus."

Finally, I am to advert to a species or variety of hæmorrhage, which seems peculiar in its character: my allusion is to those cases where the effusion recurs at stated intervals, sometimes with the exactness of well regulated menstruation, though, perhaps, rather incident to males than females—preceded nor attended by any manifestation of disease, except the sense of local fulness—nearly always issuing from the same part, and a similar quantity of blood escaping each time, the prevention or checking of which is followed by detriment to health. Examples I shall hereafter adduce, of such effusions taking place in many of the organs and structures of the body.

To exhibit a complete view of hæmorrhage, it were required to notice also those sanguineous extravasations to which occluded cavities are liable. But these have been separated as distinct diseases, and, agreeably to established usage, they will be considered by me under the heads of apoplexy, &c.

Tracing the etiology of hæmorrhage, it will be perceived, that there is the greatest liability to it about the age of puberty, when a redundancy of blood exists. As on the completion of an edifice, there is left a surplus of materials to be disposed of, so, on the cessation of the growth of the body, an excess of blood remains, sometimes removed in this way. But the tendency of hæmorrhage to its several localities, seems to be considerably influenced by the stages of life. Childhood and adolescence are prone to effusions from the nose,—for sometime afterwards they proceed from the lungs,—in middle age, the alimentary canal and the brain afford the outlet—and, from the kidneys or bladder, they are most frequently met with at a still later period. But since, in the progress to maturity, the different parts of the body may be unequally developed, and as, subsequently, changes take place from disease, or otherwise, having a similar effect, many exceptions will be found to this general rule. Nevertheless, peculiar conformations of structure no doubt predispose to particular hæmorrhages. Thus, among other instances, the short neck, and large head, invite to epistaxis or apoplexy—while the narrow, ill-shaped chest, does equally so to hæmoptysis. Certain persons, too, are singularly liable to the disease, and from different portions of the body, in whom nothing is discernible in the exterior configuration to afford an explanation. Nor is it uncommon for whole families to be thus distinguished, and who, in some instances, seem to derive the peculiarity by inheritance. An unusual want of tone in the coats of the extreme vessels, or some anomaly in their arrangement,—by which their capacity is less to resist the occasional momentum in the movements of the blood,—has here been suspected. Cases of this sort, are more frequent in scrofulous habits, where laxity in the structure of the vessels usually exist, but without any proof of such diathesis, it occurs occasionally—the hæmorrhage spontaneously bursting forth, or following the slightest wound, even an incision of the gum, or a cut of the finger, and persisting so obstinately as to prove very difficult. or not at all, to be restrained. Numerous instances of the kind, are recorded—some of which are collected in Andral's work on Pathological Anatomy, and in an Essay on the subject, by Dr. Reynell Coates, in the North American Medical Journal.

As conducive to an increased proclivity to hæmorrhagic occurrences, much is ascribed to an habitual fullness of body, and particularly where the circulation is redundant with rich blood. That such a state tends to this effect, cannot be denied, though the reverse is scarcely less true, or that it is frequently incident to attenuated habits, and where the blood is scanty and impoverished. Each condition leads to it, provided the balance in the circulation is prone to subversion, it being, under these circumstances, the consequence of unequal concentrations of blood, rather than of its quantity or quality.

But other pathological states, in which the blood undergoes still greater changes,—its consistency being so completely broken down, as to be rendered a very thin fluid, easily escaping through the exhalents,—conduce, also, to this event, and independently of any previous local accumulations. Blood, of this sort is met with in scurvy, low malignant fevers, and various other diseases, or as the effect of accidental inanition, or a penurious diet, depressing passions, the want of fresh air, or external exercise, or of solar heat and light, or whatever else affects the nerves more immediately concerned in the secretory and nutritive processes. Certain articles, taken into the system, seem especially to exercise a sort of specific operation in causing such a state of the blood. The experiments of Starke show that living long on sugar produces it, and we have reason to suppose, that the exorbitant or continued use of the neutral salts, particularly common salt, as well as the alkalies, have the same tendency. As regards common salt, we see a striking proof of it in the production of scurvy with blood of this kind. The contrary, I know, has lately been maintained by Stephens, whose authority is to be respected. It is, also, very speedily occasioned by some poisons taken internally, and by the bites of numerous venomous serpents, and even by the stings of several insects. The foregoing are what I mean by *passive* hæmorrhage.

To the causes mentioned, others are to be added of a nature accidental, accessary, or exciting, as whatever, indeed, is calculated to invigorate or quicken the circulation, or to direct or concentrate its force on any one part or organ of the body. The most prominent of these are:—

1st. Extreme heat, the operation of which is witnessed on the first accession of the warmth of spring or during, the intense heat of summer, and most conspicuously in persons who carry on their occupations in close stove-rooms, or work immediately over fires. It acts here, in the first place, merely as a stimulant accelerating the circulation, and, secondly, by relaxing the integuments which support the extreme vessels.

2d. Cold to the surface. The mode of its action is different under opposite circumstances. Being suddenly applied, as in the shower or plunging bath, a vast shock is given to the system, and a corresponding impetus to the circulation with a centripetal direction. But where the application is gradual, there is an accumulation of susceptibility, and a reaction, with febrile excitement, on an exposure to heat, or any other stimulant.

3d. Diminution in the weight or density of the atmosphere. The effect of it is illustrated chiefly in the ascent of elevated positions, and which, I am aware, has been imputed merely to inordinate exertion. We are, however, told by De Saussure, who went to the top of Mont Blanc, the highest point of the Alps, that in a state of rest, among other effects which he experienced from the rare atmosphere, the blood gushed from his nose, ears, and gums; and which is fully corroborated by Baron Humboldt, by whom the mountains of South America were ascended to their utmost pinnacle. It is, perhaps, wholly ascribable to deficient atmospherical pressure.

4th. Exertions inordinately violent, and other excitants, conduce to the same end,—as running, leaping, fighting, lifting heavy weights, or eating or drinking in excess, or vomiting, coughing, or sneezing, or bent positions of the head or body, or ligatures retarding the return of the blood, or the suppression of discharges from issues, or setons, or ulcers, the repercussion of cutaneous eruptions, or vehement gusts of passion, or emotions of any sort, or ardent venereal desires kept from indulgence, &c. Caused, however, in some of these modes, the case might not, perhaps, come within the definition of spontaneous or vital hæmorrhage, which, as I have stated, must be independent of external violence.

5th. Hæmorrhage may be owing to various morbid conditions of parts from which it immediately proceeds, or as consequential on similar states of other and remoter organs. Many of the lesions of the lungs operate to this effect, those of the heart still more, and of the liver, spleen, kidneys, and uterus, in nearly an equal degree. It is plain, that the circulation in the main vessels of an organ being impeded, the capillaries of it must become distended with blood, promotive of effusions.

6th. Gastric and intestinal irritation, especially from the habit of constipation, exerts a similar influence by inviting an excess of blood to the parts, and that of the mucous surface of the lungs from inhalations of acrid particles, or by other irritants, among which are an elongation of the uvula, enlargement of the tonsils, and ulceration of the fauces, by an extension of irritation to the pulmonary tissue.

7th. Further, it may be remarked, that hæmorrhage is prone to the shifting of position, and thus bursts forth in another part—occasioned by the sudden suppression of sanguineous discharges from artificial interference, or by the course of blood being elsewhere solicited by a higher degree of irritation, creating a predisposition to such an afflux, as among other instances, on checking the nasal, uterine, and particularly the hæmorrhoidal effusion, hemoptysis, hematemesis, or apoplexy, has speedily ensued. This is the hæmorrhage from metastasis, which, on some occasions, is very profuse.

Each of the preceding states of hæmorrhage is obviously referrible to some positive lesion. But this is not so clear in relation to those habitual discharges to which I have just alluded, so regular in their repetitions, and salutary in their tendencies. These seem to be owing to some original, inherent, constitutional idiosyncracy, by which a particular organ is made, as it were, an emunctory, to drain off a pernicious excess of blood.

No disease can be mistaken for hæmorrhage, and hence it were superfluous to say a word on the signs of discrimination. It may, however, be useful in a practical view, to indicate the means by which the different pathological states of the affection itself are to be recognised. Even here a very few remarks will suffice. Let it be recollected, as a general guide, that, in the most active form, there is the evidence of a strong febrile circulation, with redundant florid blood, abounding in crassamentum, and readily coagulating: in the less active, the reverse in a great degree of these appearances, it being thinner, dark coloured, and seldom firmly clotted,—while, in the passive, to extreme debility is united either ensanguineousness, or the blood exhibits a vast excess of serum, or otherwise is more fluid and black.

Not a little may be learned in speculating on the probable issue of the case from what has been previously said. The circumstances heretofore pointed out as influencing the event, should be carefully regarded. But, above all, we must attend to the pathological condition by which the effusion is excited and maintained. Excepting the emission be into occluded cavities, an active hæmorrhage may mostly be deemed critical and salutary, and can only operate otherwise, by excess at the moment, or by too long prevalence. There is ordinarily integrity of constitution, and relief is afforded to an oppressive degree of repletion. For the most part, different are the effects of the weaker hæmorrhages. No further expenditure of blood is here scarcely ever admissible.

An absolutely passive hæmorrhage, is always to be dreaded, it denoting a condition of things from which recoveries seldom take place. Chronic hæmorrhages, as arising usually from some permanent irritation, are also of evil import—not so much, however, on account of their copiousness, as the organic lesion of which they are the effect.

The anatomical characters of hæmorrhages, as revealed on dissection, are considerably modified by the causes, and the seats of the affection, the gradations of its activity, or its passive nature, and by the duration of the attack. These considerations, with the summary I am now to offer, will suggest a general notion of their appearances, and on coming to the description of the special hæmorrhages, I shall describe them more precisely.

An acute hæmorrhage, when profuse, sometimes leaves the tissue from which it has emanated in a natural condition, the pre-existing congestion or inflammation having been removed by the draining out of the blood, or owing to the same cause, it may be preternaturally pallid and flaccid. Generally, however, the pathological conditions mentioned, are met with in various degrees,—sometimes the phlogosis is intense and diffused, sometimes only stellated or streaked,—while, on other occasions, the vessels are merely turgid, or ecchymosed blotches, of different sizes, are discoverable. It has been said, that similar lesions are pretty constantly found in the brain, though not the seat of the effusion, and which might be presumed from the convulsions and other cerebral affections, caused by excessive losses of blood. Experiments, in which animals were bled to death, have indeed shown, that the tissues of that organ, as well as those of other structures, and particularly the mucous surfaces, are almost invariably engorged, and hence a doubt may arise, how far such a state be the cause or consequence of the hæmorrhagic effusion.

Chronic hæmorrhages more uniformly present the phenomena of inflammation, or its effects, in the thickening, or other changes of texture, with diverse other structural derangements, considerably influenced by the character of the organ affected. Not much is known of the lesions of the solids in passive hæmorrhage,—the most notable alterations in these cases being in the circulating fluids, as previously indicated. But they may be conjectured from the nature of the diseases on which they are dependent.

In entering on the examination of the pathology of hæmorrhage, it is proper to state that it can be correctly viewed only as an effect of a pre-existing anormal condition, which, in reality, constitutes the disease. Diversified as the causes of it are, they unite so far as to excite an irritation in a part, invitatory of an afflux of blood,—and the congestion thus created, is usually the immediate precursor to the effusion, and, perhaps, may be deemed its proximate cause. But effusion not happening, phlogosis is apt to be set up, and then, a hæmorrhage may ensue, as one of the incidents to the inflammatory process.

Essentially in the same mode, do some of the less active hæmorrhages take place. Local congestion or inflammation, and occasionally too, of considerable intensity, may here exist, with general debility—while in other, and more frequent instances, without either of these states being appreciable, great laxity of system is displayed,—the case then constituting the atonic hæmorrhage of the old writers. Considering those periodical effusions of blood, which, from long habit, seem to become of the nature of a natural discharge, as a hæmorrhage, they are to be embraced in the same view, since, evidently, irritation and fulness are antecedents to the effusion, very analogous to what happens in menstruation.

Of the active forms of this affection, in its several gradations, such is the rationale. Existing, however, under the opposite circumstances, of extreme depression of the vital forces, the emission of blood is usually without either of the pre-existing conditions mentioned, flowing out, as it were, by a mere leakage of the exhalents, or congestion ever happening, it is *hypostatic*, proceeding merely from gravitation of blood into the more depending parts of the body, neither of which can hardly be considered as operations of life.

Genuine or vital hæmorrhage, it is now admitted, takes place by anastamosis. No recent writer of any authority adheres to the ancient notion of the rupture of vessels, though it is true, that there are some who still credit the conjecture of the transudation of the blood. But this latter hypothesis receives, at present, no general countenance, and the doctrine of exhalation may be deemed amply entertained. This view, which was originally suggested by Morgagni, is very ably vindicated by the celebrated Bichat, whose arguments I shall cite from his work.*

He observes,—1st. "That in no instance, where he has opened the bodies of those who have died of vital hæmorrhage, has he discovered any traces of rupture of vessels, though he employed the nicest care in washing and macerating the surfaces, and examining them with a microscope.†

2d. "That in squeezing the mucous surface of the uterus, in women who have died in hæmorrhage, a number of small drops of blood may be pressed out, which manifestly correspond to the extremities of the exhalent vessels.

3d. "That hæmorrhages sometimes take place from surfaces, as the skin, where the blood indubitably comes from the exhalents, and which renders it probable that the same is the case with the mucous membranes.

4th. "That if rupture always preceded hæmorrhage, the internal surface of the womb would be a mere collection of cicatrices, as we must suppose one or more ruptures to occur at every monthly period of the catamenia.

5th. "That if in active hæmorrhages, where there is evidently a previous congestion of blood, we should admit the possibility of a rupture, how can we suppose it to take place in passive hæmorrhage, where the powers of the vessels, almost destroyed by disease, permit the blood to pass freely from their orifices.

6th. "That it is difficult to reconcile many of the phenomena of hæmorrhage, such as the extreme rapidity with which it is sometimes produced,—its appearance in another part, when it has disappeared from that in which it previously existed, and its subjection to the influence of sympathy,—with the supposition that it is produced by rupture.

7th. "The irregularity of the appearance of the blood in some hæmorrhages,—its copious flow in one instant, and its complete cessation the next, and so alternately, many times, in the course of a short period,—are difficult to be accounted for on the principle of rupture, for we must suppose the wound to be opened and closed again at every alternation of the discharge.

8th. "Comparing hæmorrhages allowedly proceeding from rupture, with others, they do not resemble them either in their phenomena or duration—they are independent of all influence from sympathy and the passions, which have considerable effect on the common kinds."

[To be continued.]

* Anatomie Generale.

† By Marendel, a late writer of high authority, who has, with infinite care, made similar examinations to a great extent, this statement is fully confirmed, and such seems now to be admitted as a demonstrated fact.

